# Positive psychological well‐being in women with obesity: A scoping review of qualitative and quantitative primary research

**DOI:** 10.1002/osp4.605

**Published:** 2022-04-25

**Authors:** Heather E. Conradson, K. Alix Hayden, Shelly Russell‐Mayhew, Shelley Raffin Bouchal, Kathryn King‐Shier

**Affiliations:** ^1^ Faculty of Nursing University of Calgary Calgary Alberta Canada; ^2^ Libraries & Cultural Resources University of Calgary Calgary Alberta Canada; ^3^ Counselling Psychology Werklund School of Education University of Calgary Calgary Alberta Canada; ^4^ Faculty of Nursing Department of Community Health Sciences University of Calgary Calgary Alberta Canada

**Keywords:** obesity, positive psychological wellbeing, women

## Abstract

**Background:**

Positive psychological well‐being (PPWB) is generally associated with improved physical health, mental well‐being, and healthy behaviors. However, it is not clear how PPWB differs in women with obesity or if improving PPWB will improve their health. The objective of this study was to summarize the evidence on PPWB in women with obesity.

**Method:**

A scoping review was conducted in APA PsycINFO, EMBASE, MEDLINE, Cochrane Central Register of Controlled Trials, CINAHL, SocINDEX, Family & Society Studies Worldwide, ProQuest Dissertations and Theses Global databases. Primary research studies, with an analysis of adult women with a BMI ≥30 kg/m^2^ with measures of PPWB are included.

**Results:**

Thirty‐two studies encompassing >57,000 women with obesity, measured constructs of PPWB included: self‐esteem, life satisfaction, positive affect, social support, vitality, happiness, self‐acceptance, and optimism. Most studies showed that PPWB was lower in women with obesity although this association dissipated in studies when health and negative social factors were considered. Improvements in PPWB were associated with weight loss and with successful lifestyle changes with and without weight loss. Positive psychological interventions (PPIs) were used to bolster psychological well‐being. PPIs were associated with improved measures of self‐esteem and well‐being.

**Conclusions:**

Prospective longitudinal and intervention studies are required to understand how evaluating and fostering PPWB might support gender‐informed obesity care.

## 1 INTRODUCTION

There is an association between positive psychological well‐being (PPWB) and improved health in general, yet the role of PPWB in improving health for women with obesity, is not clear. Positive psychological well‐being (PPWB) is associated with improved physical health, mental well‐being, and healthy behaviors.^1^ While some researchers find women with obesity have lower psychological well‐being,^2^ others dispute this association.^3^ Women with obesity disproportionately face poorer health,^4^, ^5^ poorer mental well‐being,^6^ weight discrimination,^7^ and demotivated health behaviors.^8^, ^9^, ^10^, ^11^ Enhancing PPWB may improve positive health behaviors and buffer the effects of negative self‐beliefs and negative health outcomes in women with obesity^9^, ^12^, ^13^ but the evidence to support this is limited. A systematic scoping review of the literature is needed to identify and describe the primary research on PPWB and women with obesity.

Positive psychology is the study of well‐being, and flourishing is the goal of therapy.^14^ From this perspective, well‐being is not the absence of negative function (e.g., depression, loneliness) but rather the presence of positive functions.^15^ Positive psychologists aim to support people to feel good about themselves, to feel fulfilled, and ultimately to flourish within their own unique circumstances.^14^ The goal of interventions in positive psychology is to nurture strengths rather than repair weaknesses.^16^

PPWB is a term used to describe positive psychological attributes studied in positive psychology. For the purpose of this scoping review, PPWB is defined as a multi‐dimensional construct that includes the domains of positive emotions, positive relationships, positive characteristics, and positive functions.^14^, ^17^ Specifically, the constructs included in this review are: gratitude, optimism, self‐compassion, positive relationships, engagement, self‐esteem, happiness, self‐love, self‐acceptance, accomplishment, vitality, resilience, body acceptance, body love, purpose, and meaning.^14^, ^17^

PPWB is associated with higher psychological functioning,^16^ lower incidence of chronic diseases (e.g., gastrointestinal disease, arthritis),^18^ lower risk of cardiovascular events,^19^ and improved health behaviors such as regular exercise, restful sleep, and a decreased sedentary behavior.^1^ In cardiovascular (CV) research, certain aspects of PPWB (e.g., optimism, purpose, happiness) are associated with decreased CV morbidity and mortality in those with and without CV disease.^20^, ^21^ The American Heart Association asserts that PPWB should be considered an independent factor as current data strongly suggest a causal role in better cardiovascular health.^20^

PPWB is thought to improve health through three pathways: biological (e.g., increased endorphins decrease blood pressure), behavioral (e.g., increased ability to engage in health behaviors such as regular sleep, exercise, and/or nutritious eating), and psychosocial (e.g., increased engagement of social support and self‐regulation to buffer stress and reduce negative stress hormones).^2^ It is speculated that there is a bidirectional relationship between healthy lifestyle behaviors and positive wellbeing (e.g., exercise makes you feel good and when you feel good its easier to exercise).^1^

Although all people may benefit from higher elements of PPWB (e.g., self‐esteem, resilience, self‐compassion), women with obesity differ from men with obesity. In women, a higher BMI predicts lower general psychological well‐being but not in men.^2^ Women's appraisal of their body image and quality of life are also negatively impacted by obesity.^11^, ^22^, ^23^ Women with obesity are particularly at risk for having poorer overall health, socioeconomic status, education, and employment compared to non‐obese women or men.^9^, ^24^ These differences are thought to be related to gendered cultural norms as well as weight stigmatization and discrimination.^2^, ^9^ Women with obesity experience higher rates of weight discrimination than men.^7^ Weight discrimination is associated with an increased risk of negative physical, mental, and social health and even morbidity.^9^, ^25^ With these notable differences in women and an awareness that sex and gender differences in general affect health outcomes,^26^ research that focus on women with obesity is warranted.

A scoping review was undertaken to map the available research on PPWB and women with obesity to provide an overview of the current evidence.^27^ The objective of this scoping review was to investigate what is known about PPWB in women classified as obese. More specifically, what constructs of PPWB were measured; how they were measured; what types of research methods were used to examine PPWB and what did they find; what positive psychological interventions (PPIs) were used to improve well‐being, health behaviors or general health; and lastly to identify what gaps exist in research that require further study.

## 2 METHODS

The Joanna Briggs Institute (JBI) guidelines for conducting scoping reviews informed the conduct of this review. PRISMA‐ScR reporting guidelines were used to report our findings.^27^, ^28^, ^29^, ^30^ The JBI scoping review guidelines outline the following as components of a scoping review: the review question articulated with the mnemonic PCC (population, concept, context), a protocol (available on request), a comprehensive and reproducible search strategy, a source of evidence selection, data extraction, an analysis of evidence, and the presentation of results. Three steps were used in the search strategy for this review. First, a discovery search of Google Scholar was conducted using key studies, which were then analyzed to identify key words and subject headings from titles and abstracts. Second, this analysis provided a foundation for the development of a comprehensive search in APA PscycInfo (OVID). Search development was led by a professional health sciences librarian (KAH). The full search strategies are available in the Appendix. The search was then adapted for the following databases: EMBASE (OVID), Medline and Epub Ahead of Print, In‐Process & Other Non‐Indexed Citations and Daily (OVID), Cochrane Central Register of Controlled Trials (OVID), CINAHL Plus with Full Text (Ebsco), SocINDEX with Full Text (Ebsco), Family & Society Studies Worldwide (Ebsco), and ProQuest Dissertations and Theses Global. Finally, all of the reference lists of the included studies were searched for additional studies.^27^ The original search was conducted in August of 2019 and was updated December 2020. Another update followed in December 2021, when in addition to searching for any new studies from that year, the original search was re‐run adding the terms flourish, meaning and accomplishment.

### 2.1 Inclusion and exclusion criteria

Inclusion criteria were primary research using any method (i.e., quantitative, qualitative, mixed methods), written in English, and in which PPWB measures were used with adult females (≥18 years of age) with obesity (BMI ≥ 30 kg/m^2^). The term obesity is used to describe a weight category (BMI ≥ 30 kg/m^2^) not a disease. The context of the research was open to women with obesity in different geographical, health care, or sociocultural settings. Studies in which males were included had to present specific results for the female cohort. No date restrictions were specified, and all years included in each database were searched. Exclusion criteria were articles that were not academically peer reviewed (e.g., opinion, popular culture, self‐help materials), conference abstracts, books/book chapters, editorials texts, or studies that focused on pregnant women.

## 3 RESULTS

**FIGURE 1:**
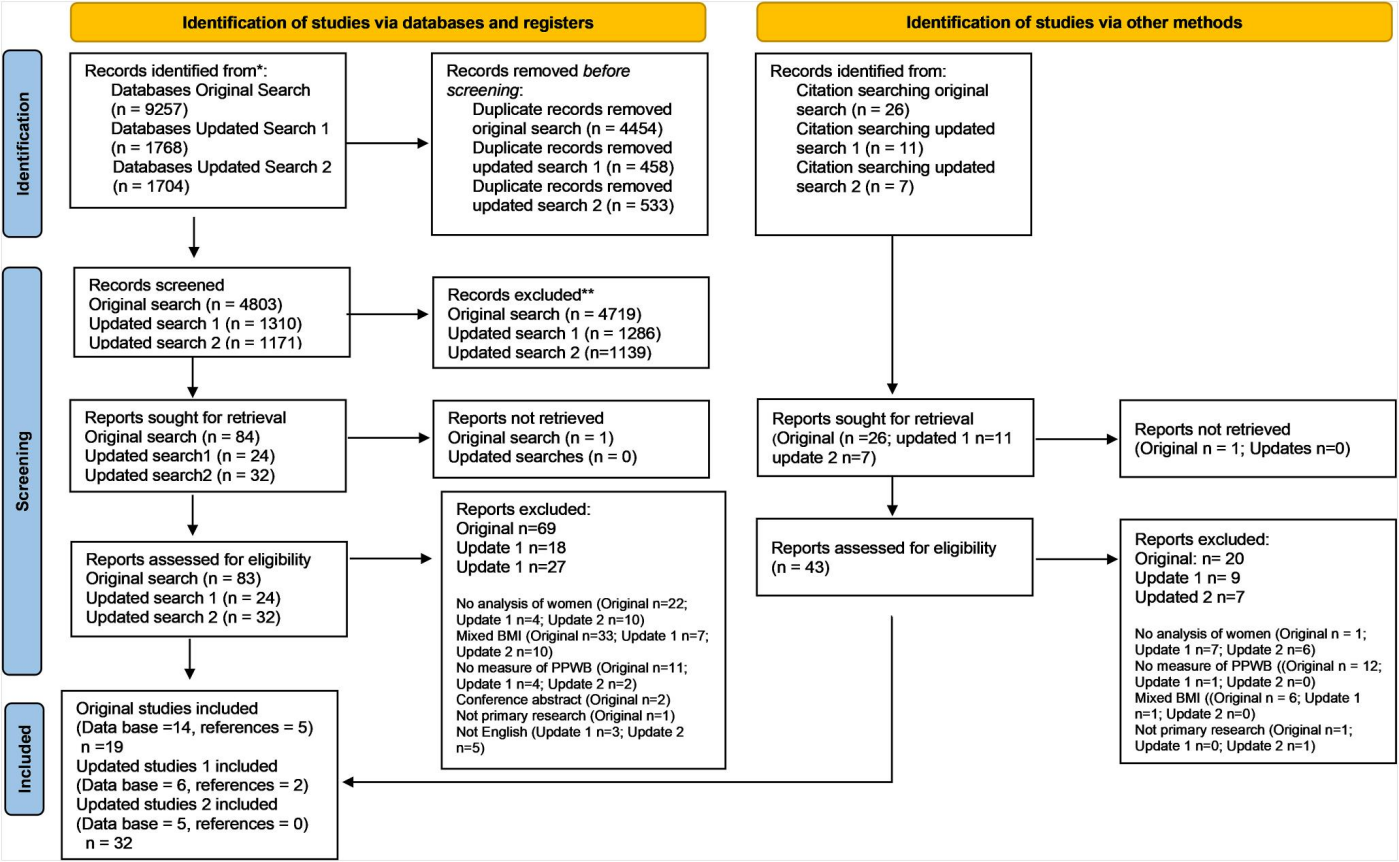
PRISMA‐SrR flow diagram

The original (August 2019) and updated (December 2020 and 2021) searches yielded 12,729 records; after duplicates were removed, 7284 records remained. Using the inclusion and exclusion criteria, two authors (HC, KKS) independently screened the records by title and abstract using Covidence^TM^ software.^31^ This tool allowed each reviewer to track and ‘vote’ on each record. Any disagreement (*n* = 154, 98% agreement) on inclusion was resolved through discussion. Once this initial process was complete, the full text articles were uploaded (*n* = 139) to Covidence^TM^ and screened for inclusion. This resulted in 25 included studies. Then references from the 25 studies were reviewed and seven more studies that met the inclusion criteria were identified. Thirty‐two studies were thus included in this review (PRISMA‐ScR diagram^32^ Figure 1). A data extraction form was prepared (by HC and KKS) to assist with summarizing relevant details from each article. Studies were read and reread to map and group evidence. Extraction was initially undertaken by HC then reviewed, edited, and verified by KKS. Study characteristics are illustrated in Table 1.

**TABLE 1:** Data extraction

First Author setting discipline	Participants	Design and positive wellbeing outcomes	Positive wellbeing measures	Results	Comment
Acmaz, G. (2013) Turkey Medicine	*N* = 133 women (n = 47 control; n = 86 with polycystic ovary syndrome (PCOS) sub‐groups: n = 35 Hirsutism‐acne (HA), *n* = 22 infertility and, n = 26 obesity Age: Control 27.8 (SD 6.5), HA 26.1 years (SD 5.0), infertility 24.3 years (SD 5.0), obesity 26.0 years (SD 6.58) Mean BMI: Control 23.4 kg/m^2^ (SD 3.1), HA 24.5 kg/m^2^ (SD 2.8), infertility 24.4 kg/m^2^(SD 3.5), obesity 33.6 kg/m^2^ (SD 2.6) Recruited from a polyclinic and control from unknown group	Design: Cross‐sectional survey comparing aspects of health in groups of women grouped by PCOS symptoms and women with no illness. Outcomes: Self‐esteem, vitality Attrition: N/A	Rosenberg self‐esteem scale (RSES) SF‐36: vitality	Women with PCOS and obesity rated their self‐esteem lower than other women with PCOS only and the control group (*p* < 0.001). All women with PCOS had lower vitality compared to the control group (*p* < 0.001).	Women with PCOS, regardless of their sub‐group reported lower vitality when compared with a healthy control group. Women with PCOS and obesity were more likely to rate their self‐esteem low compared to others with PCOS only or controls.
Ahmed, H. (2018) Iraq Medicine	*N* = 40 women Age: 25–35 years Mean BMI: 43 kg/m^2^ (range 40–50 kg/m^2^) Recruited from surgical candidates having sleeve gastrectomy	Design: Prospective longitudinal with 12‐month follow‐up Outcomes: Elements of QOL Attrition: none	QOL five‐point Likert scale: Self‐esteem, happiness, time with friends	All QOL measures were improved at 12‐month follow‐up including: Self‐esteem, spending time with friends, and feeling happy (*p* = 0.034).	One year after sleeve gastrectomy and subsequent weight loss, women reported improved self‐esteem, feeling happy and spending time with friends.
Ahmed, H. (2019) Iraq Medicine	*N* = 80 women Age: 20–39 years Mean BMI: 36 kg/m^2^ (range 31–39.9 kg/m^2^) Recruited from a private hospital and private clinic	Design: Non‐randomized descriptive longitudinal study with 6‐month treatment of either n = 40 Atkins diet or n = 40 gastric balloon. 12‐month follow‐up Outcomes: Elements of QOL Attrition: None	QOL five‐point Likert scale: Self‐esteem happiness, time with friends	All participants reported feeling happier, improved self‐esteem, spending time with friends. The gastric balloon group lost more weight than the Atkins diet group (*p* < 0.001) had greater improvements in feeling happy *p* = 0.005, improved self‐esteem *p* = 0.059 and spending more time with friends, *p* = 0.02.	PPWB measures including happiness, self‐esteem, and spending time with friends improved significantly in all women who lost weight with either gastric balloon insertion or Atkins. However, the gastric balloon group lost more weight than the Atkins group and had greater improvements in PPWB measurements.
Bacon, L. (2005) USA Nutrition	*N* = 78 women Age: 30–45 years Mean BMI: Health at Every size (HAES) group 35.9 kg/m^2 (^SD 4.6), Mean BMI: Diet group 36.7 kg/m^2^ (SD 4.2) Recruited from community	Design: RCT* HAES versus diet program; treatment included 24, 90‐min, weekly sessions plus 6 monthly optional sessions. 2‐year follow‐up Outcomes: Self‐esteem Attrition: HAES 10/39 Diet group 16/39	RSES	HAES group: Increased self‐esteem (*p* < 0.001). Diet group: Decreased self‐esteem (*p* = 0.028)	With no significant weight loss in either group, women in the HAES group improved self‐esteem while the diet groups self‐esteem worsened.
Ball, K. (2004) Australia Exercise and Nutrition	*N* = 7865 women (n = 519 with obesity) who completed 1996 survey. Age: 18–23 years BMI: ≥ 30 kg/m^2^ Recruited from urban and rural communities	Design: Cross‐sectional survey from the Women's health Australia longitudinal study. 1996 survey data. Outcomes: Life satisfaction Attrition: N/A	Life satisfaction survey: Achievements in work/career/study; family relationships; partner/close relationships; friendships; social activities.	Women with obesity reported less satisfaction in work/career/study (Odds Ratio (OR) = 0.79, confidence interval (CI) 0.66‐0.94), family relationships (OR = 0.82, CI 0.68‐0.98), partner relationships (OR = 0.55, CI 0.46‐0.65), and social activities (OR = 0.69, CI 0.58‐0.83), relative to women in the healthy weight group. There was no difference in satisfaction with friendships (OR = 1.0, CI 0.83‐1.19).	Women with obesity reported lower life satisfaction scores in work/career/study, family and partner relationships, and social activities when compared to women in healthy weight category. Friendships did not differ.
Berman, M. (2016) USA Psychiatric medicine	*N* = 21 women, with major depressive disorder Age: 23–66 years Mean BMI: 37 kg/m^2^ (range 31–50 kg/m^2^) Recruited from community	Design: Longitudinal study with a pre‐ post‐treatment and 3‐month follow‐up; 11 weekly 2‐h group sessions of self‐acceptance‐based treatment (participants broken up into 2 groups, n = 11 and n = 10). Outcomes: Well‐being, body image acceptance Attrition: 3/21	Obesity related well‐being (ORWELL‐97), body image acceptance and Action questionnaire (BIAAQ)	Self‐acceptance intervention improved: Obesity‐related well‐being (*p* < 0.001) and body image acceptance (*p* < 0.001) post‐treatment. Improvements were maintained at 3‐month follow‐up.	A self‐acceptance‐based treatment, accept Yourself, improved well‐being in women with obesity and major depressive disorder.
Böckerman P. (2014) Finland Economics/Applied science and mental health	*N* = 2245 (women = 1134 with obesity = unknown) Age: 30–54 years BMI ≥30 kg/m^2^ Recruited from health 2000 survey of Finish people	Design: Cross‐sectional survey of subjective well‐being (SWB) with full time working people outcomes: SWB Attrition: N/A	Single SWB question with a 11‐point Likert sale: “All things considered how satisfied have you been with your life as a whole during the past 30 days?”	Obesity was associated with a decrease in subjective well‐being (*p* < 0.05) and this association disappeared when health status and functional capacity were added to the model.	Lower SWB was associated with obesity yet when health and functional capacity were considered this association disappeared.
Bookwala, J. (2008) USA Psychology	*N* = 3251 (women = 1588 with obesity = 373) Age: 25‐74 BMI: ≥30 kg/m^2^ Recruited from the national survey of Midlife development in the USA (MIDUS I)	Design: Cross‐sectional survey of sex differences BMI and PWB Outcomes: Measured six domains of well‐being and combined them for a single composite measure Attrition: N/A	Ryff's scale. (Positive relations with others, self‐acceptance, autonomy, personal growth, mastery, and purpose in life)	Having a higher BMI was associated with lower psychological well‐being in women (*p* < 0.001) but not men *p* = 0.906.	Women classified as obese had lower psychological well‐being when compared to women in the normal weight category. Men psychological well‐being did not differ significantly between BMI groups.
Borkoles, E. (2016) England Psychology/Exercise Physiology	*N* = 62 women (non‐dieting lifestyle intervention n = 31, waitlist control n = 31) Age: 24–55 years BMI: >35.0 kg/m^2^ Recruited from community	Design: RCT* examining 12‐week non‐dieting lifestyle intervention followed by 40‐week maintenance phase versus waitlist control; 1‐year follow‐up Outcomes: General well‐being, self‐perception, self‐esteem Attrition: 15/31 intervention, 15/31 wait‐list control	General well‐being Schedule (GWB), self‐perception profile (SPP) measure of self‐esteem	Significant improvement in well‐being (29.9%, *p* < 0.001), and self‐perceptions (no data).	A non‐dieting lifestyle intervention improved well‐being and self‐perceptions from baseline to end of treatment, and over the 40‐week follow‐up
Brown, W.J. (2000) Australia Human Movement/Gender and Health/Psychology/Population Health	*N* = 14,762 women (n = 742 with obesity who completed 1996 surveys) Age: 18–23 years BMI: >30 kg/m^2^ Recruited from urban and rural communities	Design: Cross‐sectional survey from the Womens health Australia longitudinal study. 1996 survey data Outcomes: Mental wellbeing. Attrition: N/A	SF‐36: vitality	Women classified as overweight and obesity reported lower vitality when compared to women classified as normal or underweight. (*p* < 0.05).	Young Australian women with obesity reported lower vitality than peers classified as normal or underweight.
Burns, D. (2021) USA Nursing	*N* = 87 women with obesity Age: 30–44 years BMI: ≥30 kg/m^2^ Recruited from Facebook groups targeted 30–44‐year‐old Latino women	Design: Cross‐sectional survey examining associations between self‐esteem, body dissatisfaction and the effect on internalized weight stigma (IWS). Outcomes: Self‐esteem Attrition: N/A	RSES	When self‐esteem decreased by one unit (4‐point scale), body dissatisfaction increased by 0.270 (7‐point scale) (*p* < 0.001).Both self‐esteem (*p* < 0.001) and body dissatisfaction (*p* < 0.008) predicted IWS.	Lower self‐esteem is associated with a higher body dissatisfaction in Latino women with obesity. Self‐esteem and body dissatisfaction were predictors of IWS.
Crerand, C. (2007) USA Medicine/Psychology	*N* = 123 women (diet intervention n = 84, non‐dieting program n = 39) Mean age: 44.3 years diet intervention, 43.9 years non‐dieting program Mean BMI: 36.2 kg/m^2^ diet intervention, 35.5 kg/m^2^ non‐dieting group Recruited from community	Design: RCT* Meal replacement or balanced deficit diet (LEARN program for weight control), versus non‐dieting program (*Self‐Esteem Comes in all Sizes*), with weekly meetings for the first 20 weeks lead by psychologist and RD) and biweekly for the following 20 weeks. Outcomes: Self‐esteem Attrition: Not reported	RSES	There was an improvement in all participants' self‐esteem (*p* < 0.001) with no significant differences between groups at either the 20‐ or 40‐week follow‐up.	There was overall improvement in self‐esteem. Group assignment did not influence improvement.
Godoy‐Izquierdo, D. (2020) Spain Psychology	*N* = 100 (60 women, with 69 classified as overweight and 31 with obesity) Age: 19–57 years Mean BMI: Overweight 26.6 kg/m^2^ obese 33.5 kg/m^2^ Recruited from two primary care medical setting.	Design: Cross‐sectional survey Outcomes: Positivity and SWB (reported with happiness scale) Attrition: N/A	8‐Item positivity scale Single happiness question with a 11‐point Likert scale: “How happy are you at the present?”	No differences in positivity scores between overweight or obese participants. Obese individuals had significantly lower happiness scores (*p* = 0.014). BMI did not predict happiness after body satisfaction (*p* = 0.12 and positivity (*p* = 0.000) were considered. Self‐stigma explained variance in happiness (*p* < 0.001) but not when positivity included in model.	Women and men with obesity had significantly lower happiness scores compared to people who were overweigh. However, individuals with obesity who had higher positivity traits and higher body satisfaction were more likely to be happy. Authors suggest that positivity may supress the effects of self stigma.
Groh, C. (2015) USA Nursing	*N* = 106 African American women Age: 19–64 years Mean BMI: 41.2 kg/m^2^ (8.3) Recruited from a nurse managed center for underinsured and primary care clinic for uninsured in large urban center	Design: Longitudinal study. Repeat measures intervention (24‐week) 12 weeks of exercise, educational sessions, scripture reading followed by 12 weeks with no intervention. 12 & 24‐week follow‐up Outcomes: Subscales of SF‐36: Vitality Attrition: 51/106	SF‐36v2: vitality	Improvements from base line to 24 weeks were found in vitality (*p* < 0.001) after interventions.	Improvement in vitality after the 12‐week program. Gains declined 12 weeks post‐intervention, yet improvements remained significant.
Hill, A.J. (1998) England Psychiatry	*N* = 179 women Age: 18–75 years BMI: >30.0 kg/m^2^ Recruited from subscribers to a magazine for women size 16+	Design: Cross‐sectional survey with women divided into three obesity categories assessing predictors of psychological distress Outcomes: Self‐esteem Attrition: N/A	RSES with added questions on social life	Self‐ esteem decreased as weight increased (*p* < 0.001). Lower self‐esteem (*p* < 0.001) and peer relationships (*p* < 0.01) scores predicted lower mental health inventory scores regardless of weight category.	Self‐esteem decreased as weight increased. Yet, BMI alone did not predict mental health scores; low self‐esteem and social relationships predicted lower mental health scores.
Jorm, A. (2003) Australia Medicine/Psychology	*N* = 6919 (n = 574 women and n = 492 men with obesity) Age: Groups: 20–24 years n = 2280, 40–44 years n = 2334, 60–64 years n = 2305 BMI: >30.0 kg/m^2^ Recruitment from a random sample of participants from a longitudinal community study (PATH through life Project).	Design: Cross‐sectional survey of three age groups and four weight categories (under weight, acceptable, overweight, & obese). Outcomes: positive affect measures. Attrition: N/A	PANAS	Women with obesity had lower positive affect (*p* < 0.001). However, when physical health, vigorous activity, negative support from family, years of education, and financial problems were controlled the association was not significant.	Women with obesity had lower positive affect than women classified as acceptable weight. This association was gone after controlling for negative physical and social factors.
Laferrere, B. (2002) USA Medicine	*N* = 145 women Mean age: 46.3 ± 11.1 years Mean BMI: 35.2 ± 4.2 kg/m^2^ Recruited from middle class urban community	Design: Cross‐sectional survey of pre and post menopausal African american (AA) n = 80 and white women (W) n = 65. Outcomes: Medical outcomes study Short (MOS‐SF‐36): Survey includes life satisfaction, self‐esteem, vitality, social activity. Attrition: N/A	SWLS RSES SF‐36:Vitality	No difference between AA and W women self‐esteem or life satisfaction. Yet there were differences between pre and post menopausal women pre‐menopause women had less vitality (*p* = 0.001), life satisfaction (*p* < 0.01).	No difference in life satisfaction and self‐esteem were found between African american and white women with obesity. Post‐menopausal AA women had more vitality and life satisfaction compared to pre‐menopausal AA women.
Latif, E. (2014) Economics Canada	*N* = 28,952 (n = 6006 women, n = 5837 with obesity) Mean age: women 46.7 years, men 44 years BMI: Obesity ≥30.0 kg/m^2^ obesity Recruited from the Canadian national Public survey 1994‐2006	Design: Cross‐sectional survey Outcomes: Happiness Attrition: N/A	Single happiness question with 5 ordered choices: So unhappy in life, very unhappy, somewhat unhappy, somewhat happy, and happy in life	Obesity is associated with a reduction in happiness in women between the ages of 25‐54 (*p* = 0.04). Obesity was not associated with happiness in men. Statistical model controlled for age, marital statis, education, income, urban location, own home, and provinces.	Obesity is associated with reduced happiness in women between the ages of 25‐54. Obesity is not associated with happiness in men.
Mensinger, J. (2016) USA Psychology/Population health	*N* = 80 women Age: 30–45 years BMI: 30–45 kg/m^2^ Recruited from community	Design: RCT* comparing 6‐month group‐based weight ‐neutral (HUGS program) or weight‐loss interventions with 2‐year follow‐up Outcome: Self‐esteem Attrition: Weight‐loss: 6‐month 7/40 24‐ month 19/40, weight‐neutral: 6‐month 1/40 24‐month 21/40	RSES	Both groups increased self‐esteem (*p* < 0.001)	Women in both interventions significantly increased their self‐esteem.
Pasco, J. (2013) Australia Medicine/Psychiatry	*N* = 273 women (n = 68 with obesity) Age: 29‐84 BMI: ≥30 kg/m^2^ Recruited from the Geelong Osteoporosis study from general population	Design: Cross‐sectional survey Outcomes: positive and negative affect Attrition: N/A	PANAS	No association between BMI category and positive affect. Obesity was associated with high negative affect (OR 1.95 CI 0.57‐1.69) and physical illness explained this association.	Women's positive affect was not associated with their BMI category.
Polat, H. (2020) Turkey Nursing	*N* = 90 women Mean age: 32.98 ± 7.74 years Mean BMI: 40.10 ± 5.14 kg/m^2^ Recruited from a University hospital dietitian department	Design: Cross‐sectional Outcome: Self‐esteem Attrition: N/A	RSEM	Women with obesity had moderate levels of self‐esteem. Self‐esteem was higher in those who were physically active (*p* < 0.05). Positive association between self‐esteem and sexual quality of life (*p* < 0.01).	Obese women had moderate levels of self‐esteem. Exercise enhanced self‐esteem. High self esteem was associated with improved sexual quality of life in women with obesity.
Ripsch, J. (2002) USA Social work PhD Thesis	*N* = 13 women Age: 32–53 years Mean BMI: 37 kg/m^2^ Recruitment from a snowball sampling from a University and community.	Design: Grounded theory Women with obesity who reported positive sense of self on the Orientation to life questionnaire) were interviewed. Outcomes: Theory development on how women maintained a positive sense of self Attrition: none	Orientation to life questionnaire	Participants chose to have a positive sense of self irrespective of their weight. They shared supportive relationships with friends, family and/or mentors, developed skills and competencies, cared for others, and engaged in positive self talk.	Women with obesity attributed their positive sense of self to interpersonal connections, positive self‐talk as well as their skills and abilities.
Rodino, I. (2016) Australia Psychology	*N* = 403 women (n = 73 with obesity) Age: 34.5 years (SD = 4.8) BMI: >30 kg/m^2^ Recruited from an infertility clinic	Design: Cross‐sectional survey examining wellbeing of women with and without polycystic ovary syndrome Outcomes: positive affect and self‐esteem Attrition: 445/1000 participants returned survey	PANAS RSES	Women with obesity exhibited lower levels of self‐esteem as compared to their normal weight and overweight counterparts (*p* < 0.001). Higher self‐esteem was associated with a higher positive affect (p < 0.001).	Women with obesity scored lower in self‐esteem. Higher self‐esteem was associated with a higher level of positive affect.
Sarwer, D (2013) USA Medicine/Psychiatry	*N* = 390 women (n = 311) Mean age: 51.5 (SD = 11.5) BMI: 30.0–50.0 kg/m^2^ Recruited from six primary care practices	Design: RCT* with three arms: usual care, brief lifestyle counseling (BLC) or enhanced LC (ELC). Women were followed for 2 years. Outcomes: Self‐esteem Attrition: 20/130 usual care, 19/130 B LC, 15/129 ELC	Impact of weight on quality of life‐Lite (IWQOL)	A mean population weight loss of 3.7 kg ± 0.4% at 12 months and 3.0 ± 0.4% at 24 months was associated with improved self‐esteem (*p* < 0.001) in women.	Women's self‐esteem measures improved significantly with modest weight reduction regardless of treatment stream,
Sato, K. (2020) Japan Political science	*N* = 6359 women and men in China/USA (n = 1380/4979). Number of women with obesity not reported. Mean age: China/USA women 46.1/50.5 years, men 45.5/49.0 years BMI: ≥30 kg/m^2^ Recruited between 2009 and 2013	Design: Cross‐sectional survey subjective well‐being (happiness) Outcome: Happiness Attrition: N/A```	Single happiness question with 11‐point Likert scale: “ overall, how happy would you say you are currently?”	No significant association between obesity and happiness in women overall. Obese men in China were happier (*p* < 0.001) than obese men in USA.	There was no association with obesity and happiness in women from China and USA.
Smith (2014) USA Nursing	*N* = 68 women Age: 18–24 years Mean BMI: 35.6 (5.1) kg/m^2^ Recruited from a community healthcare center	Design: Cross‐sectional survey of young women with obesity Outcomes: Self‐esteem Attrition: N/A	RSES	Self‐esteem had a strong inverse association with stress (r = − 0.6, *p* < 0.001), and loneliness (r = −0.69, *p* < 0.001). Women who rated their health poor/fair had lower self‐esteem (*p* = 0.001) compared to those who rated their health good/very good.	Poor self‐esteem was associated with stress, and loneliness in women with obesity. Poor self‐esteem was also associated with personal assessment of poor/fair health.
Toft B. (2020) Denmark Lifestyle Rehabilitation/Nursing	*N* = 10 (n = 5 women, n = 5 men) Age: 30–69 years BMI: 40–48 kg/m^2^ Recruited from a lifestyle intervention hospital program	Design: Qualitative Hermeneutic. Interviews were conducted 18 months after a 12‐month health promotion intervention by an interdisciplinary team Outcome: Barriers and facilitators of well‐being Attrition: one woman	Well‐being defined by womens descriptions of elevated mood and vitality	Women participants appreciated how the process of vitality gave them the desire and courage to stay committed to healthy living. Vitality came from reducing self‐blame, strengthening confidence and a strong sense of self.	Women found increasing their pride, confidence and self‐acceptance improved their mood and vitality which enabled them to engage in an active everyday life.
Ul‐Haq, Z. (2014) UK Public health	*N* = 163,066 (54.5% women) n = 39,941 classified obese Age: 30–72 years BMI: > 30.0 kg/m^2^ Recruited form biobank participants	Design: Cross‐sectional Outcomes: Happiness Attrition: N/A	Single happiness question with a 6‐chocies: “In general, how happy are you?”	Women with obesity had greater odds of being unhappy when compared to normal weight women (OR: Obesity Class l ‐ 1.27 (CI 1.16‐1.40 *p* < 0.001), Class ll‐ 1.41 (CI 1.23‐1.61, *p* < 0.001), Class lll‐ 2.11 (1.79‐2.49, *p* < 0.001). There was no association between happiness and obesity when self‐reported health (SRH) was included in the model	Women with obesity reported more unhappiness compared to those of normal weight. When adjusted for self‐reported health, the association between women with obesity and lower ratings of happiness disappeared. Author suggested self‐reported health may be a mediator of happiness.
Unlu, S. (2019) Turkey Nutrition and Dietetics	*N* = 250 women (80 in each category normal, overweight, and obese) Age: 18–64 years BMI: Obese group ≥30 kg/m^2^ Recruited from a family healthcare center	Design: Cross‐sectional Outcomes: Self‐esteem Attrition: N/A	Self‐esteem Inventory	Women with obesity were 12.19 times more likely to have lower low self‐esteem (*p* < 0.001). and 91.22 times more likely to have body dissatisfaction (*p* < 0.001) compared to the normal weight group.	Obesity negatively effects self‐esteem and body satisfaction.
Wadsworth, T. (2014) USA Sociology	*N* = 1,319,340, n = 82,116 women (33% with obesity), n = 498,224 men (32% with obesity) Mean age: Female 46.78 years, men 44.67 years BMI: Obese class I 30–34.9 kg/m^2^, class II 35–39.9 kg/m^2^, Class III >40 kg/m^2^ Corrected self‐reported weight Recruited from telephone survey across communities in the USA	Design: Cross‐sectional Behavior risk factor Surveillance System (BRFSS) survey data 2005‐2008 Outcome: Life satisfaction Attrition: N/A	Single life satisfaction question with 3 choices: “In general, how satisfied are you with your life?”	Women with class I obesity or class II or III report lower levels of life satisfaction compared to non obese women (OR 0.78, 0.57, *p* < 0.001). Although men with obesity also report lower levels of satisfaction, women were statistically lower (*p* = 0.001). Good health, being employed, white, married, and having higher education improve life satisfaction scores for women.	People with obesity report lower life satisfaction and this association is stronger for women than men.
Wee, C. (2014) USA Medicine	*N* = 337 (women/men with BMI ≥35 n = 230/107) Mean age 48.1 years BMI: ≥35 kg/m^2^ Recruited from four primary care practices in greater Boston	Design: Cross‐sectional interview/survey. Examined how specific QOL domains explained health Utility (overall well‐being) in African american, Caucasian, and Hispanic population. Outcomes: Self‐esteem Attrition: N/A	Impact of weight on quality of life‐lite (IWQOL‐lite)‐subscale self‐esteem	Self‐esteem did not explain diminished well‐being in women with obesity. Social stigma(public distress) and impaired sexual function were associated with lower well‐being (*p* < 0.05)	Self‐esteem was not associated with well‐being in African american, Caucasian, or Hispanic women with obesity.
van Zutven, K. (2015) Australia Psychology	*N* = 2734 (women/men classified non‐obese n = 1220/769, obese n = 276/169) Age 20–44 years BMI: Obese ≥30 kg/m^2^ Non‐obese 20–29.9 kg/m^2^ Recruited from 2006 Australian community survey (Food, Drink, lifestyle and wellbeing survey)	Design: Cross‐sectional survey Outcomes: Satisfaction with life Attrition: N/A	SWLS	Women with obesity had lower satisfaction with life scores than non‐obese women (<0.001). This relationship was not significant after considering health, weight and shape concerns and binge eating (*p* = 0.23).	Women with obesity had lower satisfaction with life than non‐obese participants. However, this association dissipated after considering health, weight/shape concerns, and binge eating.

### 3.1 Study characteristics

The 32 studies included an analysis of more than 57,000 women, ages 18–84 years, who had a BMI greater than or equal to 30 kg/m^2^. The studies were conducted in the United States (*n* = 11),^2^, ^33^, ^34^, ^35^, ^36^, ^37^, ^38^, ^39^, ^40^, ^41^, ^42^, ^43^, ^44^ Australia (*n* = 5),^45^, ^46^, ^47^, ^48^, ^49^, ^50^ England (*n* = 2),^51^, ^52^ Iraq (*n* = 2),^53^, ^54^ Turkey (*n* = 3),^55^, ^56^, ^57^ Canada (*n* = 1),^58^ Denmark (*n* = 1),^59^ Finland (*n* = 1),^60^ Japan (*n* = 1),^61^ Spain (*n* = 1),^62^ and United Kingdom (*n* = 1).^3^ The disciplines of lead authors were predominantly psychology and medicine. Participants were recruited from a variety of locations including health centers, universities, and both urban and rural communities.

### 3.2 PPWB constructs and measures

Self‐esteem was the most frequently measured PPWB construct and was measured in 14 of the 32 studies.^33^, ^35^, ^37^, ^38^, ^40^, ^41^, ^43^, ^44^, ^48^, ^51^, ^52^, ^55^, ^56^, ^57^ Other constructs of PPWB included: life satisfaction^37^, ^42^, ^45^, ^49^, ^60^; positive affect^47^, ^50^, ^51^, ^62^; social support^52^, ^53^, ^54^; vitality^36^, ^37^, ^46^, ^57^, ^59^; happiness^3^, ^53^, ^54^, ^58^, ^61^, ^62^; self‐acceptance^34^; and optimism.^39^ Various approaches were used to measure self‐esteem that included the Rosenberg Self Esteem Survey (RSES),^33^, ^35^, ^37^, ^38^, ^41^, ^43^, ^48^, ^52^, ^56^, ^57^ the Self Perception Profile (SPP),^51^ Self‐esteem Inventory^55^ and the self‐esteem subscale of the Impact of Weight on Quality of Life‐Lite (IWQOL) scale.^40^ There was no indication of the source of the questions used to measure self‐esteem in two studies.^53^, ^54^

Satisfaction was measured using the Satisfaction with Life scale (SWLS)^37^, ^49^ and twice with different questions. In two cases, a single question rating life satisfaction was used (question source not identified).^42^, ^60^ In another case, a group of questions rating women's satisfaction with work/career/study, family relationships, partner/close relationships, friendships, and social activities (questionnaire source not referenced) was used.^37^ Positive affect was measured using the Positive and Negative Affect Scale (PANAS)^47^, ^50^, ^51^ and the positivity scale.^62^ Social support was examined using questions about social life and friends in three studies^52^, ^53^, ^54^ (questionnaire source not referenced). Vitality was measured with the sub‐scales from the SF 36.^36^, ^37^, ^46^, ^57^ Vitality was also a theme in one qualitative study.^59^ There was no indication of the origin of the questions used to measure happiness in any studies.^3^, ^53^, ^54^, ^58^, ^61^, ^62^ Self‐acceptance was measured using the Body Image Acceptance and Action Questionnaire (BIAAQ).^34^ Lastly, optimism was measured with the Orientation to Life Questionnaire.^39^

Rather than measuring an individual construct, some researchers chose instruments that measured well‐being and included multiple constructs of PPWB.^2^, ^34^, ^51^ The Obesity Related Well‐being questionnaire (ORWELL‐97) was used to measure the impact of obesity on positive affect, social activities, physical activity, self esteem, relationships, and perception of health.^34^ The Ryff's Scale was used to measure positive relationships, self‐acceptance, autonomy, personal growth, mastery, and purpose in life.^2^ Finally, the General Well‐being Schedule (GWB) was used to measures positive well‐being, vitality, general health, anxiety, depression, and self control.^51^

### 3.3 Research methods and findings

Study types varied and included interventional studies (5 randomized controlled trials (RCT)^33^, ^35^, ^38^, ^40^, ^51^ and 4 longitudinal studies^34^, ^36^, ^53^, ^54^), 21 cross‐sectional surveys,^3^, ^10^, ^37^, ^41^, ^42^, ^43^, ^44^, ^45^, ^46^, ^47^, ^48^, ^49^, ^50^, ^52^, ^55^, ^56^, ^57^, ^58^, ^60^, ^61^, ^62^ and 2 qualitative studies.^39^, ^59^

### 3.4 Cross‐sectional surveys

Women's ratings of PPWB varied. Cross‐sectional study results are illustrated in Table 2. Most studies found women with obesity rated their self‐reported PPWB significantly lower than men or women without obesity.^2^, ^42^, ^45^, ^46^, ^48^, ^52^, ^55^, ^57^, ^58^ While some researchers found the association between lower PPWB and obesity was attenuated when other variables (e.g., self‐health, body satisfaction, positivity, social factors) were included in the statistical model.^3^, ^47^, ^49^, ^60^, ^62^ Still some researchers found no association between obesity and aspects of PPWB (positive affect, happiness, self‐esteem).^44^, ^50^, ^61^ Though PPWB was significantly lower for women with obesity overall, women who were in a sub‐cohort of those who reported higher economic status, employment, education, health, relationships, sexual quality of life, exercise levels and body satisfaction were more likely to report higher PPWB than those who were not.^55^, ^56^, ^58^, ^62^

**TABLE 2:** Summary of cross‐sectional studies' self‐ratings of PPWB outcomes in women with obesity

	Context		Comparators											PPWB measures						
Reference	General population	Health care centers	BMI <18.5 kg/m^2^	BMI 18.5–25 kg/m^2^	BMI 25–29.9 kg/m^2^	BMI ≥30 kg/m^2^	Country	Healthy volunteer	Men	Menopause	PCOS	Race	18–24 years of age	General well‐being	Happiness	Life satisfaction	Positive affect	Positive relationships	Self‐esteem	Vitality
Acmaz 2013		x			x			x			x	x				↓				↓
Ball 2004	x			x									x			↓		↓		
Böckerman 2014	x		x	x	x				x							↔				
Bookwala 2008	x			x					X					↓						
Brown 2000	x		x	x	x								x							↓
Burns 2021	x					x													↓^a^	
Godoy‐Izquierdo 2020		x		x	x				X						↓					
Hill 1998	x			x	x	x													↓	
Jorm 2003	x		x	x	x												↔			
Laferrere 2002	x									x		x				↔			↔	↑^b^
Latif 2014	x								X						↓					
Pasco 2013	x			x													⊗			
Polat 2020		x				x										⊕				
Rodino 2016		x		x	x	x		x			x								↓	
Sato 2020		x				x	x		X						⊗					
Smith 2014		x				x							x						↓^c^	
Ul‐Haq 2014	x				x										↔					
Unlu 2019		x		x															↓	
Wadsworth 2014	x					x			X							↓				
Wee 2014		x										x							⊗	
van Zutven 2015	x			x	x											↔				

Abbreviations: BMI, body mass index; PCOS‐Polycystic Ovary Syndrome. Symbols, ↓Low levels; ↓ ^a^ low Self‐esteem predicted internalized weight stigma; ↑^b^ Vitality higher in post‐menopausal women compared to premenopausal African American women with obesity; ↓^c^ lower self esteem associated with stress, loneliness, and poor health; ⊕ moderate level; ↔ negative association not significant after controlling variables; ⊗ no association.

### 3.5 Interventional studies

Aspects of PPWB were improved in all interventional studies. However, improving PPWB was not the primary focus of any study. Interventional studies are divided into two groups: RCTs and longitudinal studies. Interventional study results are shown in Table 3.

**TABLE 3:** Summary of interventions with PPWB outcomes in women

Reference	Population									Intervention									Control			PPWB and weight outcomes											
	BMI ≥30 kg/m^2^		BMI ≥35 kg/m^2^		BMI ≥40 kg/m^2^		Depression		Underserved AAW	Bariatric surgery	Lifestyle behavior		*PPI		Weight loss			Weight‐neutral	Usual care		Waitlist	General well‐being		Happiness	Healthy eating		Self‐esteem		Positive relationships	Vitality		Weight	
**RCT**																																	
Bacon 2005*	x											x		x		x	x										↑						↔
Borkoles 2016		x										x					x			x		↑					↑						↔
Crerand 2007*	x													x		x	x										↑						↓↔
Mensinger 2016*	x													x		x	x									↑	↑						↓↔
Sarwer 2013	x											x				x			x								↑						↓
**Longitudinal**																																	
Ahmen 2018				x						x													↑				↑	↑					↓
Ahmen 2019	x									x						x							↑				↑	↑					↓
Berman 2016*	x					x								x			x					↑											↔
Groh 2015	x							x				x					x														↑		

Abbreviations: BMI, body mass index; RCT, randomized Controlled Trial; AAW, African American women; * PPI, positive psychology intervention. Symbols: ↓ Weight‐loss, ↔ weight neutral, ↓↔ weight‐PPWB outcomes with both weight loss and neutral groups.

### 3.6 Randomized controlled trials

The primary purpose of all five RCTs was to examine non‐diet and/or diet treatment outcomes. Self‐esteem was measured in all five RCTs and one also measured overall well‐being. Most women who participated in these lifestyle change program reported improved PPWB irrespective of weight loss.^33^, ^35^, ^38^, ^40^, ^51^ Women in a non‐dieting lifestyle program arm of a RCT^33^ and in both the non‐diet and the diet arms of randomized controlled lifestyle trials^35^, ^38^ reported improved self‐esteem, with and without weight loss. However, women in the diet treatment of a RCT^33^ who did not sustain weight loss reported lower self‐esteem.

### 3.7 Longitudinal studies

In the four longitudinal studies, women were followed for 24–52 weeks. PPWB was examined as an outcome in two studies focused on weight loss treatment outcomes.^53^, ^54^ Researchers found that when women lost weight, regardless of the weight loss treatment, the ratings of PPWB improved. Women who lost large amounts of weight (>10–35 kg) after sleeve gastrectomy procedure,^53^ gastric balloon insertion,^54^ or adhering to an Atkins diet,^54^ reported higher self‐esteem, happiness and spending more time with friends. The greater the weight loss, the greater the gains in PPWB. PPWB was also examined as an outcome in two studies that focused on improving lifestyle behaviors and mental health outcomes.^34^, ^36^ In one instance, after a faith‐based lifestyle change program, underserved African American women reported increased vitality.^36^ While in another study, women with a major depressive disorder reported improved over all well‐being after a weight neutral lifestyle behavior program with a positive physiological intervention (Accept Yourself).^34^

### 3.8 Qualitative studies

Using a grounded theory approach, women who reported a positive sense of self, were asked how they maintained their PPWB.^39^ The participants conveyed that having a positive sense of self required supportive relationships with friends, family and mentors, the ability to develop skills and competencies, involvement in caring for others, and the ability to engage in positive self‐talk.^39^ Another researcher, using Hermeneutic inquiry, found women in a lifestyle intervention program, described improved vitality and mood came from reducing self‐blame, strengthening their confidence, and self acceptance.^59^ Women appreciated how an increase in vitality gave them the desire and courage to stay committed to healthy living and engage in their activities of everyday life.^59^

### 3.9 Positive psychological interventions (PPIs)

Positive psychological interventions (PPIs) were used to enhance psychological well‐being in four studies.^33^, ^34^, ^35^, ^38^ PPIs included a Health at Every Size (HAES) group intervention where the researchers taught women self‐acceptance, size diversity, and how healthy people come in all sizes.^33^ Other researchers used self‐esteem boosting exercises from Johnson's book *Self‐Esteem Comes in All Sizes*.^35^ While another research group used the HUGS (**H**ealth focused, **U**nderstanding lifestyle, **G**roup supported, and **S**elf‐esteem building) program to build self‐esteem.^38^ Lastly, researchers used a self‐acceptance treatment model called Accept Yourself.^34^ Although the types of PPIs varied, participants were seen weekly, for one to 2 hours, for a duration of 11–26 weeks and had follow‐up ranging from 6 months to 2 years.

Women who participated in PPIs reported improved self‐esteem or well‐being in all four‐studies.^33^, ^34^, ^35^, ^38^ Although in two studies, women who did not receive the PPI also improved their self‐esteem.^35^, ^38^ In addition to improving PPWB, women reported increased physical activity^33^, ^38^ and eating more fruit and vegetables.^38^ Women's physical health measures of LDL cholesterol (bad cholesterol)^33^, ^38^ and blood pressure were lower.^33^, ^34^ Finally, women reported higher mental well‐being measures of quality of life,^34^, ^38^ and body image acceptance^34^, ^35^ as well as less depression^34^, ^35^ and weight self‐stigma.^34^

## 4 DISCUSSION

This is the first scoping review to our knowledge that has a focus on PPWB in women with obesity. In studies that included over 57,000 women with obesity, many constructs of PPWB were measured using a variety of instruments. In the majority of studies, PPWB was lower in women with obesity. Improved PPWB was associated with weight loss and with successful lifestyle changes with and without weight loss. Although few in numbers, PPIs were associated with improved self‐esteem and well‐being. These findings offer glimpses into how PPWB may be associated with improved aspects of health, and healthy behaviors in women with obesity. Still, the causal relationships are not explained by the studies included in this scoping review. There are many knowledge gaps that need to be addressed before providers who work to support women with obesity can determine if and what role PPWB will play in their care.

There is a notable gap in the evidence of how women with obesity experience PPWB and what aspects they may or may not wish to address. For example, it is not known if women with obesity feel a need to improve aspects of their PPWB. There has been an acceleration of calls over the last decade for greater research participant engagement in the research process.^63^ Using patient/person‐oriented research, which often includes creating early partnerships with potential research participants to ensure that research questions and the methods to gain answers are relevant and the findings are interpreted in a manner that resonates with those who will benefit most from the research, women with obesity would have much to contribute and gain.

Further, researchers were not able to illuminate what constructs of PPWB are most important to measure in women with obesity. Some constructs of PPWB may have greater importance in terms of health outcomes in general populations. Cardiovascular researchers have identified that optimism, positive psychological constructs, and subjective well‐being are linked to significantly better cardiovascular outcomes, decreased rehospitalization and reduced risk of mortality.^19^, ^21^, ^64^, ^65^, ^66^ Prospective studies have yet to identify if certain constructs of PPWB are associated with better health outcomes in women with obesity specifically. Longitudinal prospective studies are needed to identify what constructs of PPWB, if any, are associated with the improved health outcomes in this particular population.

Using an instrument that measures multiple aspects of PPWB and reporting sub‐scale scores could provide a richer and more substantive understanding of the relationship between psychological well‐being and health in women with obesity. For example, the PERMA‐profiler measures five constructs of well‐being including **p**ositive emotion, **e**ngagement, **r**elationships, **m**eaning and **a**ccomplishment.^14^ It also measures subjective health, loneliness, negative emotion, happiness and provides a composite score for overall PPWB. This is one of many tools that could be used to gain greater insights into multiple aspects of PPWB and possible inter‐related variables for women with obesity.

Future research is needed to explore if women who optimized PPWB feel more able to increase their activity and/or if increasing activity bolsters their PPWB. Not surprisingly, women who reported sustained improvements in exercise also reported improved PPWB.^33^, ^38^, ^51^, ^59^ PPWB and improved health behaviors are thought to have a reciprocal relationship.^1^ As such, the interactions between exercise and PPWB in women with obesity should be investigated further.

In cross‐sectional studies, the relationship between health, PPWB and obesity in women is not clear. Health status was not measured^2^, ^43^, ^44^, ^45^, ^48^, ^55^, ^62^ or adjusted for in multi‐variable analyses.^46^, ^58^ In studies where analyses were adjusted for subjective health ratings, the association between obesity and lower PPWB was attenuated.^3^, ^42^, ^49^, ^60^ It is possible that poor health supersedes BMI as a predictor of low PPWB. These findings suggest that when measuring PPWB it would be helpful to also measure physical health.

Further research is needed to understand the relationships between psychosocial health and PPWB in women with obesity. In this review, the particular psychosocial variables that were associated with PPWB included functional capacity, self‐stigma, social‐stigma, body image, body satisfaction, social determinates of health, and social support.^34^, ^42^, ^43^, ^44^, ^45^, ^47^, ^60^, ^62^ Additional mechanisms that contribute to lower PPWB in women with obesity may include lower socioeconomic status, level of education and employment status; internal weight stigmatization; societal prejudice; and weight discrimination; and body dissatisfaction and mental health concerns.^6^, ^67^

Race was not included as a variable in the majority of studies.^35^, ^41^, ^45^, ^46^, ^47^, ^48^, ^49^, ^50^, ^52^, ^53^, ^54^, ^55^, ^56^, ^57^, ^58^, ^59^, ^60^, ^62^ Still, in the few studies for which race was reported, women of color were not included in representative proportions.^2^, ^3^, ^33^, ^34^, ^38^, ^39^, ^51^ In all but four^40^, ^42^, ^43^, ^44^ over 90% of the women were white.^2^, ^3^, ^33^, ^34^, ^38^, ^39^, ^51^ The influence of race on PPWB in women with obesity was examined in three studies.^36^, ^37^, ^43^ Research that includes race and provides representative data is needed.

With only a few studies in which PPIs were examined to improve PPWB, clinicians lack sufficient evidence to recommend them in treatment programs. Researchers suggest that positive emotions may improve negative self‐beliefs and health.^9^, ^12^, ^13^ Although HAES, HUGS, Accept Your Self, and Self‐esteem Comes in All Sizes PPIs have demonstrated associations between improved PPWB and improved health behaviors (increased exercise, healthy eating) or other outcome measures (decreased depression, blood pressure and LDL cholesterol measures), the causal relationship between improved PPWB constructs and improved health is yet to be elucidated. More RCTs are needed to assess if indeed psychological well‐being promoting interventions can improve health outcomes in women with obesity.

Taken together, the role of PPWB in supporting women with obesity remains to be determined. Prospective longitudinal and intervention studies are required to understand how fostering PPWB and evaluating relevant outcomes might support gender‐informed obesity care.

## 5 STRENGTHS AND LIMITATIONS

Significant strengths of this study were that the search was conducted by a professional health science librarian (KAH) and the authors followed a rigorous methodological and reporting framework (JBI^28^, ^29^ and PRISMA‐ScR^32^). Another strength is multiple databases were searched that encompassed a wide variety of disciplines and these databases are not often included in health reviews (i.e., SocINDEX with Full Text, Family & Society Studies Worldwide). A thoughtful preliminary literature review strategy was used to determined keywords. However, even with a comprehensive strategy, omission of potentially relevant keywords is a limitation inherent to researching multidimensional constructs. Lastly this study is limited by including English‐only language publications.

## CONFLICT OF INTEREST

The authors declare no conflict of interest.

## Final Search Strategies: Positive Psychological Well‐being in Women with Obesity

Changes to search:

- Update #1: December 2020 did not limit searches by English
- Update #2: December 2021 added in Search Concept A:
  ◦ (flourish* or accomplishment*)
  ◦ (meaning adj3 (life or making)

Database(s): **APA PsycInfo** 1806 to November Week 52,021 Search Strategy:

**Table:**

#	Searches	Results
1	exp *Well being/	37,763
2	exp Gratitude/	1497
3	exp Optimism/	4619
4	exp Self‐compassion/	1485
5	exp *Self‐esteem/	20,260
6	exp happiness/	8344
7	(optimism or optimistic).tw,id.	16,484
8	gratitude.tw,id.	3717
9	self‐love.tw,id.	423
10	self‐compassion.tw,id.	2520
11	self‐esteem.tw,id.	48,340
12	self‐accept*.tw,id.	2752
13	vitality.tw,id.	4744
14	resilience.tw,id.	30,283
15	body acceptance.tw,id.	150
16	body love.tw,id.	7
17	happiness.tw,id.	17,496
18	(flourish* or accomplishment*).tw,id.	15,832
19	(meaning adj3 (life or making)).tw,id.	10,748
20	(emotional adj2 stability).tw,id.	2970
21	((hedonic* or eudaimonia*) adj2 (well‐being or wellbeing)).tw,id.	270
22	((positive* or psycholog* or optimal) adj4 (well‐being or wellbeing)).tw,id.	23,487
23	((positive* or social) adj4 (relationships or engagement)).tw,id.	38,113
24	or/1‐23	222,451
25	exp *Obesity/	21,295
26	exp *Overweight/	22,786
27	exp *Body Weight/	47,599
28	exp *Weight Loss/	2864
29	exp *Body mass Index/	4442
30	exp *Weight Control/	4217
31	(obese or obesity).tw,id.	43,792
32	(overweight or over‐weight).tw,id.	16,519
33	(weight adj2 (excessive or management or loss)).tw,id.	14,842
34	(bmi or body mass index).tw,id.	30,301
35	or/25‐34	86,885
36	exp Human Females/	151,783
37	wom?n*.mp.	334,308
38	female*.mp.	1,125,130
39	or/36‐38	1,275,335
40	24 and 35 and 39	2509
41	(child* or adolescen* or teen*).ti.	474,138
42	40 not 41	1980
43	limit 42 to peer reviewed journal	1627

## Database(s): **Ovid MEDLINE(R) and Epub Ahead of Print, In‐Process, In‐Data‐Review & Other Non‐Indexed Citations and Daily **1946 to 14 December 2021 Search Strategy:

**Table:**

#	Searches	Results
1	exp Optimism/	898
2	*Self Concept/	26,726
3	exp Happiness/	4902
4	(optimism or optimistic).tw,kf.	16,327
5	gratitude.tw,kf.	1886
6	self‐love.tw,kf.	63
7	self‐compassion.tw,kf.	1484
8	self‐esteem.tw,kf.	22,818
9	self‐accept*.tw,kf.	851
10	vitality.tw,kf.	13,645
11	resilience.tw,kf.	34,152
12	body acceptance.tw,kf.	119
13	body love.tw,kf.	3
14	happiness.tw,kf.	8066
15	(flourish* or accomplishment*).tw,kf.	13,892
16	(meaning adj3 (life or making)).tw,kf.	3348
17	(emotional adj2 stability).tw,kf.	1102
18	((hedonic* or eudaimonia*) adj2 (well‐being or wellbeing)).tw,kf.	139
19	((positive* or psycholog* or optimal) adj4 (well‐being or wellbeing)).tw,kf.	18,299
20	((positive* or social) adj4 (relationships or engagement)).tw,kf.	22,108
21	or/1‐20	170,497
22	*Obesity/or *obesity, morbid/	153,057
23	*Overweight/	16,722
24	*Body weight/	30,407
25	*Weight loss/	16,920
26	*body mass index/	23,060
27	(obese or obesity).tw,kf.	342,038
28	(overweight or over‐weight).tw,kf.	78,878
29	(weight adj2 (excessive or management or loss)).tw,kf.	107,283
30	(bmi or body mass index).tw,kf.	280,154
31	or/22‐30	631,956
32	wom?n*.mp.	1,300,606
33	female*.mp.	9,486,632
34	Female/	9,280,090
35	or/32‐34	9,661,543
36	21 and 31 and 35	4521
37	Limit 36 to English language	4300
38	(Child* or adolescen* or teen*).ti.	952,106
39	37 not 38	3290
40	Limit 39 to (address or autobiography or bibliography or biography or clinical trial, veterinary or clinical trials, veterinary as topic or comment or dataset or dictionary or directory or editorial or “expression of concern” or government document or interactive tutorial or interview or lecture or legal case or legislation or letter or news or newspaper article or observational study, veterinary or patient education handout or periodical index or personal narrative or portrait or video‐audio media or webcast)	20
41	39 not 40	3270

## Database(s): **Embase **1974 to 2021 December 14 Search Strategy:

**Table:**

#	Searches	Results
1	*wellbeing/	11,724
2	exp *optimism/	1203
3	exp *self esteem/	4379
4	exp *happiness/	2697
5	(optimism or optimistic).tw,kw.	20,376
6	gratitude.tw,kw.	2379
7	self‐love.tw,kw.	90
8	self‐compassion.tw,kw.	1617
9	self‐esteem.tw,kw.	29,082
10	self‐accept*.tw,kw.	1054
11	vitality.tw,kw.	18,653
12	resilience.tw,kw.	38,196
13	body acceptance.tw,kw.	128
14	body love.tw,kw.	2
15	happiness.tw,kw.	9485
16	(flourish* or accomplishment*).tw,kw.	16,185
17	(meaning adj3 (life or making)).tw,kw.	3850
18	(emotional adj2 stability).tw,kw.	1381
19	((hedonic* or eudaimonia*) adj2 (well‐being or wellbeing)).tw,kw.	131
20	((positive* or psycholog* or optimal) adj4 (well‐being or wellbeing)).tw,kw.	22,718
21	((positive* or social) adj4 (relationships or engagement)).tw,kw.	26,088
22	or/1‐21	189,895
23	*obesity/or *morbid obesity/	202,407
24	*body weight/	33,088
25	*body weight loss/	7821
26	*body mass/	36,120
27	(obese or obesity).tw,kw.	503,836
28	(overweight or over‐weight).tw,kw.	118,812
29	(weight adj2 (excessive or management or loss)).tw,kw.	170,887
30	(bmi or body mass index).tw,kw.	505,017
31	or/23‐30	989,459
32	wom?n*.mp.	1,777,395
33	female*.mp.	10,443,381
34	female/	10,162,538
35	or/32‐34	10,643,932
36	22 and 31 and 35	5897
37	(child* or adolescen* or teen*).ti.	1,092,546
38	36 not 37	4754
39	limit 38 to conference abstracts	1491
40	38 not 39	3263
41	limit 40 to (book or book series or major reference work or trade journal)	3
42	40 not 41	3260

## Database(s): **EBM Reviews ‐ Cochrane Central Register of Controlled Trials **November 2021 Search Strategy:

**Table:**

#	Searches	Results
1	exp Optimism/	0
2	self concept/	2504
3	exp Happiness/	226
4	(optimism or optimistic).tw,kw.	1221
5	gratitude.tw,kw.	329
6	self‐love.tw,kw.	8
7	self‐compassion.tw,kw.	691
8	self‐esteem.tw,kw.	3738
9	self‐accept*.tw,kw.	104
10	vitality.tw,kw.	2666
11	resilience.tw,kw.	2313
12	body acceptance.tw,kw.	21
13	body love.tw,kw.	0
14	happiness.tw,kw.	1166
15	(flourish* or accomplishment*).tw,kw.	520
16	(meaning adj3 (life or making)).tw,kw.	310
17	(emotional adj2 stability).tw,kw.	247
18	((hedonic* or eudaimonia*) adj2 (well‐being or wellbeing)).tw,kw.	18
19	((positive* or psycholog* or optimal) adj4 (well‐being or wellbeing)).tw,kw.	7692
20	((positive* or social) adj4 (relationships or engagement)).tw,kw.	2045
21	Or/1‐20	22,678
22	obesity/or obesity, morbid/	13,728
23	Overweight/	5623
24	Body weight/	8747
25	Weight loss/	6566
26	body mass index/	10,861
27	(obese or obesity).tw,kw.	46,798
28	(overweight or over‐weight).tw,kw.	18,310
29	(weight adj2 (excessive or management or loss)).tw,kw.	24,227
30	(bmi or body mass index).tw,kw.	66,465
31	or/22‐30	111,332
32	wom?n*.mp.	172,398
33	female*.mp.	865,885
34	female/	479,414
35	or/32‐34	910,661
36	21 and 31 and 35	1233
37	(child* or adolescen* or teen*).ti.	92,605
38	36 not 37	1049

## Database(s): **CINAHL Plus with Full Text**

**Table:**

#	Query	Limiters/Expanders	Results
S1	(MM “Psychological Well‐Being”)	Search modes ‐ find all my search terms	13,748
S2	(MM “Optimism”)	Search modes ‐ find all my search terms	1160
S3	(MM “Self Concept”)	Search modes ‐ find all my search terms	14,631
S4	(MM “Happiness”)	Search modes ‐ find all my search terms	2296
S5	TI ((meaning N3 (life or making)) OR AB ((meaning N3 (life or making))	Search modes ‐ find all my search terms	3723
S6	TI ((gratitude or self‐love or self‐compassion or self‐esteem or self‐accept* or optimism or optimistic)) OR AB ((gratitude or self‐love or self‐compassion or self‐esteem or self‐accept* or optimism or optimistic))	Search modes ‐ find all my search terms	23,720
S7	TI ((vitality or resilience or “body acceptance” or “body love” or happiness or flourish* or accomplishment*)) OR AB ((vitality or resilience or “body acceptance” or “body love” or happiness or flourish* or accomplishment*))	Search modes ‐ find all my search terms	29,513
S8	TI (emotional N2 stability) OR AB (emotional N2 stability)	Search modes ‐ find all my search terms	476
S9	TI (((hedonic* or eudaimonia*) N2 (well‐being or wellbeing))) OR AB (((hedonic* or eudaimonia*) N2 (well‐being or wellbeing)))	Search modes ‐ find all my search terms	83
S10	TI (((positive* or psycholog* or optimal) N4 (well‐being or wellbeing))) OR AB (((positive* or psycholog* or optimal) N4 (well‐being or wellbeing)))	Search modes ‐ find all my search terms	11,446
S11	TI (((positive* or social) N4 (relationships or engagement))) OR AB (((positive* or social) N4 (relationships or engagement)))	Search modes ‐ find all my search terms	26,839
S12	S1 OR S2 OR S3 OR S4 OR S5 OR S6 OR S7 OR S8 OR S9 OR S10 OR S11	Search modes ‐ find all my search terms	108,830
S13	(MM “Obesity”) OR (MM “Obesity, Morbid”)	Search modes ‐ find all my search terms	60,158
S14	(MM “Body Weight”)	Search modes ‐ find all my search terms	8589
S15	(MM “Weight Loss”)	Search modes ‐ find all my search terms	10,766
S16	(MM “Body Mass Index”)	Search modes ‐ find all my search terms	13,517
S17	(MM “Weight Control”)	Search modes ‐ find all my search terms	4607
S18	TI ((obese or obesity)) OR AB ((obese or obesity))	Search modes ‐ find all my search terms	112,718
S19	TI ((overweight or over‐weight)) OR AB ((overweight or over‐weight))	Search modes ‐ find all my search terms	35,331
S20	TI ((weight N2 (excessive or management or loss))) OR AB ((weight N2 (excessive or management or loss)))	Search modes ‐ find all my search terms	34,484
S21	TI ((bmi or “body mass index”)) OR AB ((bmi or “body mass index”))	Search modes ‐ find all my search terms	100,154
S22	S13 OR S14 OR S15 OR S16 OR S17 OR S18 OR S19 OR S20 OR S21	Search modes ‐ find all my search terms	220,164
S23	(MH “female”)	Search modes ‐ find all my search terms	2,113,616
S24	TI (women or woman or female*) OR AB (women or woman or female*)	Search modes ‐ find all my search terms	652,525
S25	S23 OR S24	Search modes ‐ find all my search terms	2,250,278
S26	S12 AND S22 AND S25	Search modes ‐ find all my search terms	2707
S27	S12 AND S22 AND S25	Search modes ‐ find all my search terms	2707
S28	S12 AND S22 AND S25	Limiters ‐ Scholarly (peer reviewed) Journals Search modes ‐ find all my search terms	2598
S29	TI (child* or adolescen* or teen*)	Search modes ‐ find all my search terms	396,398
S30	S28 NOT S29	Search modes ‐ find all my search terms	1985

## Database(s): **SocIndex with Full Text**

**Table:**

#	Query	Limiters/Expanders	Results
S1	TI ((meaning N3 (life or making)) OR AB ((meaning N3 (life or making)) OR KW ((meaning N3 (life or making))	Search modes ‐ find all my search terms	3380
S2	TI ((gratitude or self‐love or self‐compassion or self‐esteem or self‐accept* or optimism or optimistic)) OR AB ((gratitude or self‐love or self‐compassion or self‐esteem or self‐accept* or optimism or optimistic)) OR KW ((gratitude or self‐love or self‐compassion or self‐esteem or self‐accept* or optimism or optimistic))	Search modes ‐ find all my search terms	19,044
S3	TI ((vitality or resilience or "body acceptance" or "body love" or happiness or flourish* or accomplishment*)) OR AB ((vitality or resilience or "body acceptance" or "body love" or happiness or flourish* or accomplishment*)) OR KW ((vitality or resilience or "body acceptance" or "body love" or happiness or flourish* or accomplishment*))	Search modes ‐ find all my search terms	23,365
S4	TI (emotional N2 stability) OR AB (emotional N2 stability) OR KW (emotional N2 stability)	Search modes ‐ find all my search terms	556
S5	TI (((hedonic* or eudaimonia*) N2 (well‐being or wellbeing))) OR AB (((hedonic* or eudaimonia*) N2 (well‐being or wellbeing))) OR KW (((hedonic* or eudaimonia*) N2 (well‐being or wellbeing)))	Search modes ‐ find all my search terms	98
S6	TI (((positive* or psycholog* or optimal) N4 (well‐being or wellbeing))) OR AB (((positive* or psycholog* or optimal) N4 (well‐being or wellbeing))) KW (((positive* or psycholog* or optimal) N4 (well‐being or wellbeing)))	Search modes ‐ find all my search terms	1294
S7	TI (((positive* or social) N4 (relationships or engagement))) OR AB (((positive* or social) N4 (relationships or engagement))) OR KW (((positive* or social) N4 (relationships or engagement)))	Search modes ‐ find all my search terms	27,824
S8	TI ((obese or obesity)) OR AB ((obese or obesity)) OR KW ((obese or obesity))	Search modes ‐ find all my search terms	6233
S9	TI ((overweight or over‐weight)) OR AB ((overweight or over‐weight)) OR KW ((overweight or over‐weight))	Search modes ‐ find all my search terms	2420
S10	TI ((weight N2 (excessive or management or loss))) OR AB ((weight N2 (excessive or management or loss))) OR KW ((weight N2 (excessive or management or loss)))	Search modes ‐ find all my search terms	1342
S11	TI ((bmi or “body mass index”)) OR AB ((bmi or “body mass index”)) OR KW ((bmi or “body mass index”))	Search modes ‐ find all my search terms	3734
S12	TI (women or woman or female*) OR AB (women or woman or female*) OR KW (women or woman or female*)	Search modes ‐ find all my search terms	260,073
S13	S1 OR S2 OR S3 OR S4 OR S5 OR S6 OR S7	Search modes ‐ find all my search terms	72,303
S14	S8 OR S9 OR S10 OR S11	Search modes ‐ find all my search terms	9756
S15	S12 AND S13 AND S14	Search modes ‐ find all my search terms	195
S16	TI (child* or adolescen* or teen*)	Search modes ‐ find all my search terms	146,392
S17	S15 NOT S16	Search modes ‐ find all my search terms	158
S18	S15 NOT S16	Limiters ‐ Scholarly (peer reviewed) Journals Search modes ‐ find all my search terms	145

## Database(s): **Family & Society Studies Worldwide**

**Table:**

#	Query	Limiters/Expanders	Results
S1	TI ((meaning N3 (life or making)) OR AB ((meaning N3 (life or making)) OR KW ((meaning N3 (life or making))	Search modes ‐ find all my search terms	1772
S2	TI ((gratitude or self‐love or self‐compassion or self‐esteem or self‐accept* or optimism or optimistic)) OR AB ((gratitude or self‐love or self‐compassion or self‐esteem or self‐accept* or optimism or optimistic)) OR KW ((gratitude or self‐love or self‐compassion or self‐esteem or self‐accept* or optimism or optimistic))	Search modes ‐ find all my search terms	17,917
S3	TI ((vitality or resilience or "body acceptance" or "body love" or happiness or flourish* or accomplishment*)) OR AB ((vitality or resilience or "body acceptance" or "body love" or happiness or flourish* or accomplishment*)) OR KW ((vitality or resilience or "body acceptance" or "body love" or happiness or flourish* or accomplishment*))	Search modes ‐ find all my search terms	14,276
S4	TI (emotional N2 stability) OR AB (emotional N2 stability) OR KW (emotional N2 stability)	Search modes ‐ find all my search terms	383
S5	TI (((hedonic* or eudaimonia*) N2 (well‐being or wellbeing))) OR AB (((hedonic* or eudaimonia*) N2 (well‐being or wellbeing))) OR KW (((hedonic* or eudaimonia*) N2 (well‐being or wellbeing)))	Search modes ‐ find all my search terms	28
S6	TI (((positive* or psycholog* or optimal) N4 (well‐being or wellbeing))) OR AB (((positive* or psycholog* or optimal) N4 (well‐being or wellbeing))) KW (((positive* or psycholog* or optimal) N4 (well‐being or wellbeing)))	Search modes ‐ find all my search terms	1346
S7	TI (((positive* or social) N4 (relationships or engagement))) OR AB (((positive* or social) N4 (relationships or engagement))) OR KW (((positive* or social) N4 (relationships or engagement)))	Search modes ‐ find all my search terms	17,765
S8	TI ((obese or obesity)) OR AB ((obese or obesity)) OR KW ((obese or obesity))	Search modes ‐ find all my search terms	34,659
S9	TI ((overweight or over‐weight)) OR AB ((overweight or over‐weight)) OR KW ((overweight or over‐weight))	Search modes ‐ find all my search terms	14,494
S10	TI ((weight N2 (excessive or management or loss))) OR AB ((weight N2 (excessive or management or loss))) OR KW ((weight N2 (excessive or management or loss)))	Search modes ‐ find all my search terms	9381
S11	TI ((bmi or “body mass index”)) OR AB ((bmi or “body mass index”)) OR KW ((bmi or “body mass index”))	Search modes ‐ find all my search terms	27,191
S12	TI (women or woman or female*) OR AB (women or woman or female*) OR KW (women or woman or female*)	Search modes ‐ find all my search terms	283,222
S13	S1 OR S2 OR S3 OR S4 OR S5 OR S6 OR S7	Search modes ‐ find all my search terms	50,753
S14	S8 OR S9 OR S10 OR S11	Search modes ‐ find all my search terms	56,046
S15	S12 AND S13 AND S14	Search modes ‐ find all my search terms	552
S16	TI (child* or adolescen* or teen*)	Search modes ‐ find all my search terms	326,804
S17	S15 NOT S16	Search modes ‐ find all my search terms	469
S18	S15 NOT S16	Limiters ‐ Scholarly (peer reviewed) Journals Search modes ‐ find all my search terms	425

## **Database(s):** PROQUEST DISSERTATIONS

((noft(optimism OR optimistic OR gratitude OR self‐love OR self‐compassion OR self‐esteem OR self‐accept* OR vitality OR resilience OR "body acceptance" OR "body love" OR happiness or flourish* or accomplishment*) OR noft(meaning WITHIN‐3 (life or making)) OR noft(emotional WITHIN‐2 stability) OR (noft(hedonic* WITHIN‐2 well‐being) OR noft(hedonic* WITHIN‐2 wellbeing) OR noft(eudaimonia* WITHIN‐2 well‐being) OR noft(eudaimonia* WITHIN‐2 wellbeing)) OR (noft(positive* WITHIN‐2 well‐being) OR noft(positive* WITHIN‐2 wellbeing) OR noft(psycholog* WITHIN‐2 well‐being) OR noft(psycholog* WITHIN‐2 wellbeing) OR noft(optiimal WITHIN‐2 well‐being) OR noft(optimal WITHIN‐2 wellbeing)) OR (noft(positive* WITHIN‐2 relationships) OR noft(positive* WITHIN‐2 engagement) OR noft(social WITHIN‐2 relationships) OR noft(social WITHIN‐2 engagement))) AND noft(obese OR obesity OR overweight OR over‐weight OR bmi OR "body mass index") AND noft(women OR woman OR female*)) NOT ti((child* OR adolescen* OR teen*))
